# Ferroptosis Induction Improves the Sensitivity of Docetaxel in Prostate Cancer

**DOI:** 10.1155/2022/4930643

**Published:** 2022-05-24

**Authors:** Xiumei Chen, Yang Yu, Sudong Liang, Zhenghui Guan, Hui Shi, Qingyi Zhu, Jianzhong Lin

**Affiliations:** ^1^Department of Geriatrics, The First Affiliated Hospital of Nanjing Medical University, Nanjing, China; ^2^Department of Urology, Shanghai General Hospital of Nanjing Medical University, Shanghai, China; ^3^Department of Urology, Taizhou Clinical Medical School of Nanjing Medical University, Nanjing, China; ^4^Central Laboratory, Jiangsu Health Development Research Center, Nanjing, China; ^5^Department of Urology, The Second Affiliated Hospital of Nanjing Medical University, Nanjing, China

## Abstract

Docetaxel resistance seriously affects its clinical application in prostate cancer (PCa). Ferroptosis is a type of iron-dependent cell death driven by lipid peroxidation. It has been recently found that ferroptosis influences various biological processes. However, the potential role of ferroptosis in docetaxel chemotherapy for PCa is still elusive. In this study, we aimed to explore whether altering the level of ferroptosis can affect docetaxel sensitivity in PCa. The results indicated that docetaxel promoted ferroptotic cell death in several PCa cells, and ferroptosis inducers, erastin, and RSL3 markedly increased the cytotoxic effect of docetaxel. Furthermore, our results showed that ferroptosis resistance was closely associated with docetaxel insensitivity in PCa-resistant cells. Erastin or RSL3 rendered resistant PCa cells susceptible to docetaxel, with elevated levels of lipid ROS and decreased protein expression of GPX4 and SLC7A11. Moreover, treatment with erastin and RSL3 led to significant suppression of resistant tumors, and the combination of RSL3 with docetaxel significantly halted tumor growth in vivo when compared with either drug. Taken together, our findings indicate that ferroptosis is involved in docetaxel resistance, and its inducers are promising therapeutic strategies for advanced PCa.

## 1. Introduction

Prostate cancer (PCa) is currently the most frequently diagnosed cancer and the important leading cause of cancer-related deaths in the United States and Europe [1]. Docetaxel is generally approved as a first-line chemotherapeutic agent for the treatment of advanced PCa; however, the efficacy of docetaxel is severely limited by the development of resistance. Although several factors have been demonstrated to promote docetaxel resistance, strategies for targeting them are still not satisfactory. New involved mechanisms and treatment options are urgently needed to be identified for improving docetaxel effectiveness for such patients.

Ferroptosis is a recently identified mode of an iron-dependent programmed cell death characterized by excess reactive oxygen species-induced lipid peroxidation. Emerging evidence suggests that many cancer cells are vulnerable to ferroptosis including lymphoma [2], cervical cancer [3], lung cancer [4], and pancreatic cancer [5]. Glutathione peroxidase (GPX4) and solute carrier family 7 member 11 (SLC7A11) are considered as two pivotal regulators of ferroptosis. Pharmacologic or genetic inhibition of GPX4 [6, 7] or SLC7A11 [8, 9] induced ferroptotic cell death is confirmed to be a promising targeted therapy strategy in a variety of tumors. Combination therapy of ferroptosis inducers (FINs) like erastin and RSL3 with other chemotherapeutic agents shows a significant synergistic effect in many cancers [10–13]. In addition, studies have confirmed that ferroptosis is involved in resistance to antiandrogen drugs, and ferroptotic induction promotes their sensitivity in PCa [14, 15]. However, the therapeutic potential of erastin and RSL3 in docetaxel resistance remains elusive in PCa.

In this study, we demonstrated that docetaxel exerted its cytotoxic effect partly by inducing ferroptosis, and ferroptosis insensitive contributed to docetaxel resistance in PCa. The higher expression of SLC7A11 and GPX4 protein and stronger resistance to their inhibitors, erastin, and RSL3 were found in docetaxel-resistant cells. The administration of them was confirmed to reverse docetaxel resistance in vitro and in vivo. Thus, our results indicate that FINs enhance the therapeutic efficacy of docetaxel and may offer a potential strategy to overcome docetaxel resistance in PCa.

## 2. Materials and Methods

### 2.1. Cell Culture and Reagents

Cell lines, LNCaP, C4-2B, 22Rv1, DU145, PC3, and VCaP were obtained from the Chinese Academy of Sciences, Shanghai Institute of Biochemistry and Cell Biology (Shanghai, China). Docetaxel-resistant cells (PC3-DR, DU145-DR, and VCaP-DR) were developed according to our previous study [16]. Briefly, resistant cell lines were cultured over 6 months by increasing the concentration of docetaxel in a stepwise manner. Docetaxel, erastin, RSL-3, and Ferrostatin-1 (Fer-1) were purchased from MCE (Shanghai, China).

### 2.2. Cell Viability Assay

Cells were seeded onto 96-well microplates, cultured at 37°C for 48 h, and treated with different drugs at indicated concentrations. Cellular viability was assessed by performing a CCK-8 assay. Values were read using a multidetector microplate reader (BioTek Instruments, Inc., USA).

### 2.3. Bioinformatics Analysis

GEPIA (http://gepia.cancer-pku.cn/), an online tool for analyzing the transcriptional and clinical data from TCGA, was used to assess the prognostic value of selected genes in PCa by dividing the TCGA cohorts into two groups according to the quartile cutoff value expression of designated genes. In addition, the RNA-seq data of six patients (GSE51005) were downloaded from Gene Expression Omnibus (GEO) database (https://www.ncbi.nlm.nih.gov/geo/). We evaluated gene differential levels from PCa biopsy specimens between before and after treatment with docetaxel chemotherapy using the DESeq2 package.

### 2.4. ROS and MDA Measurement

Cellular reactive oxygen species (ROS) generation in the supernatant of HNC cell lysates was assessed by adding 10 *μ*M 2′,7′-dichlorofluorescein diacetate (DCF-DA) (Nanjing Jiancheng Bioengineering Institute, E-004-1-1) for 30 min at 37°C. The ROS levels were analyzed with fluorometer at 520 nm. The relative MDA concentration in PCa cells was assessed using a Lipid Peroxidation (MDA) Assay Kit (A003-4-1) according to the manufacturer's instructions. Briefly, MDA reacted with thiobarbituric acid (TBA) to generate an MDA-TBA adduct and quantified colorimetrically (OD = 532 nm).

### 2.5. Glutathione (GSH) Assay

The relative GSH concentration in cells was assessed using a GSH Detection Assay Kit (Nanjing Jiancheng Bioengineering Institute, A006-2-1) according to the manufacturer's instructions. Briefly, cells were washed with PBS, centrifuged at low speed, and broken by ultrasound. The supernatant was collected and separated at 3500 rpm for 10 min. The values were measured spectrophotometrically at 405 nm.

### 2.6. Western Blotting Analysis

Cells were harvested and lysed via RIPA buffer. The protein content of the supernatant was determined using BCA Protein Assay kit (Beyotime, Shanghai, China). Equal quantities of protein were electrophoresed on 10% sodium dodecyl sulfate-polyacrylamide gels and transferred to PVDF membranes. The membranes were blocked and incubated overnight at 4°C with primary antibodies and secondary antibodies. Antibodies were as follows: anti-human SLC7A11 (Abcam, ab175186, 1 : 1000), anti-human GPX4 (Abcam, ab41787, 1 : 1000), and GAPDH (Abcam, ab181602, 1 : 1000).

### 2.7. Animal Experiments

All animal experiments were conducted in accordance with the NIH Guide for the Care and Use of Laboratory Animals, with the approval of Nanjing Medical University. Briefly, PC3-DR or VCaP-DR cells were injected into the flanks of Balb/c nude mice (male, 6–8 weeks). The tumors reached about 50–80 mm^3^ average volumes and were then randomized into different treatment groups, including vehicle (20 mL DMSO), erastin (20 mg/kg in 20 mL DMSO, i.p., daily), and RSL3 (100 mg/kg in 20 mL DMSO, i.p., biweekly). For combination therapy experiments, mice were randomized into different treatment groups, including vehicle, docetaxel (10 mg/kg, i.p., biweekly), erastin (20 mg/kg, i.p., daily) + docetaxel (10 mg/kg, i.p., biweekly), and RSL3 (100 mg/kg, i.p., biweekly) + docetaxel (10 mg/kg, i.p., biweekly). Tumor volume was monitored regularly, and the mice were killed after three weeks. The size of the tumor and the weight of the mice were measured every 3 days, and tumor volume was calculated with the following formula: *V* = (length) × (width)^2^/2.

### 2.8. Statistical Analysis

All data were obtained from three independent experiments and expressed as the mean ± standard deviation. Comparisons of the two sets of data were using an unpaired Student's *t*-test. A two-way analysis of variance (ANOVA) followed by Tukey post hoc test was used for multiple comparisons. ^∗^*P* < 0.05 was considered to indicate a statistically significant difference.

## 3. Results

### 3.1. Docetaxel Induces Ferroptotic Cell Death in Prostate Cancer Cells

**Figure 1:**
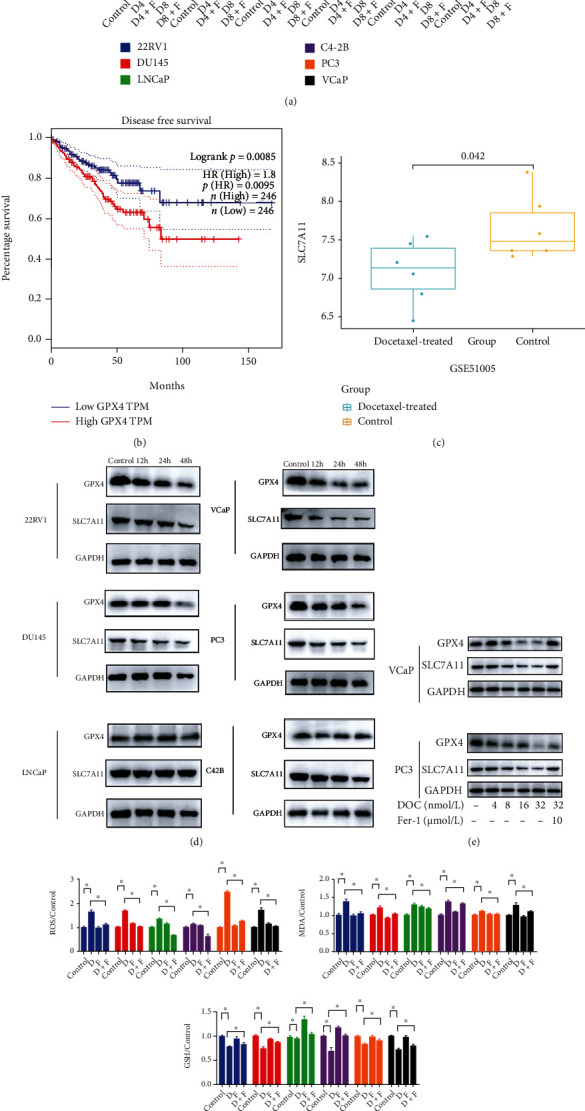
Docetaxel induces ferroptotic cell death in prostate cancer. (a) LNCaP, C4-2B, 22Rv1, DU145, PC3, and VCaP cells were treated with docetaxel (D, 4 nM or 8 nM) alone or in combination with Ferrostatin-1 (F, 5 *μ*M). CCK8 was used to assess cell viability. (b) Kaplan-Meier disease-free survival analysis with one-sided log-rank test of GPX4 gene expression in PCa patients using TCGA datasets. (c) SLC7A11 mRNA expression was analyzed after docetaxel treatment in a clinical data analysis (GSE51005). (d) After the treatment of 8 nM docetaxel at different times (12 h, 24 h, 48 h), GPX4 and SLC7A11 protein expressions in LNCaP, C4-2B, 22Rv1, DU145, PC3, and VCaP cells were examined by western blotting (WB). (e) WB was used to assess the protein level of GPX4 and SLC7A11 in PC3 and VCaP cells after exposure to 4 nM, 8 nM, 16 nM, and 32 nM docetaxel alone or in combination with Ferrostatin-1 (F, 10 *μ*M). (f) The relative contents of ROS, MDA, and GSH in LNCaP, C4-2B, 22Rv1, DU145, PC3, and VCaP cells were detected by commercial kits. The data were reported as mean ± SD. ^∗^*P* < 0.05.

To test whether ferroptosis is involved in the cytotoxic effect of docetaxel in PCa, we first treated different PCa cells (LNCaP, C4-2B, 22Rv1, DU145, PC3, and VCaP) with docetaxel alone with Fer-1, an identified ferroptosis inhibitor; the result indicated that Fer-1 significantly rescued the inhibitory effect of docetaxel on cell growth in all six PCa cells (Figure 1(a)). GPX4 and SLC7A11 are key negative regulators of ferroptosis. Kaplan-Meier analysis indicated that patients with high GPX4 levels have a markedly shorter disease-free survival period than those with low GPX4 expression in patients with PCa (Figure 1(b)). Further analysis of clinical data (GSE51005) showed that the mRNA expression of SLC7A11 was obviously decreased after docetaxel chemotherapy (Figure 1(c)). In this study, we found that their levels were significantly suppressed in a time-dependent manner after treatment of 8 nM docetaxel in 22Rv1, DU145, PC3, and VCaP cells; however, similar results were not found in LNCaP and C4-2B cells (Figure 1(d)). Furthermore, we also found that docetaxel exerted a dose-dependent inhibitory effect on their protein expression in PC3 and VCaP cells, and Fer-1 blocked docetaxel-induced suppression of GPX4 and SLC7A11 (Figure 1(e)). ROS accumulation, lipid peroxidation, and GSH depletion are regarded as critical events in ferroptosis. Our results showed that docetaxel resulted in a marked increase in the levels of intracellular ROS and oxidative stress marker malondialdehyde (MDA) and a significant decrease in GSH, which could be reversed by cotreatment with Fer-1 in PCa cells (Figure 1(f)). Taken together, these findings suggest that oxidative stress-dependent ferroptotic cell death may participate in the inhibitory effect of docetaxel on PCa cells.

### 3.2. Ferroptosis Induction Improves Sensitivity to Docetaxel in Prostate Cancer Cells

**Figure 2:**
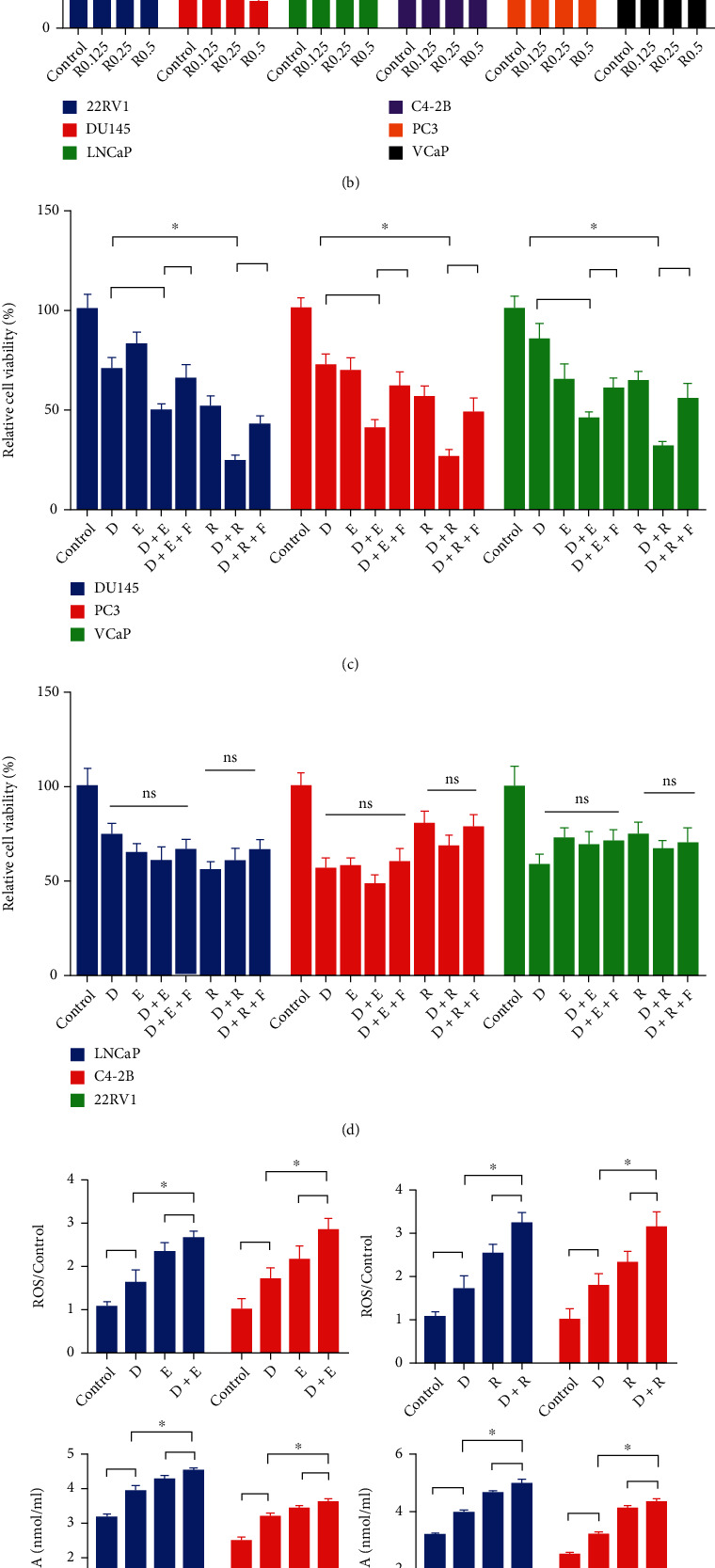
Ferroptosis induction improves sensitivity to docetaxel in prostate cancer cells. (a, b) CCK8 was used to determine cell viability in LNCaP, C4-2B, 22Rv1, DU145, PC3, and VCaP cells after the treatment of erastin (E, 7.5 *μ*M, 15.0 *μ*M, and 30 *μ*M) or RSL-3 (R, 0.125 *μ*M, 0.25 *μ*M, and 0.5 *μ*M) in different concentrations. (c, d) Cell viability was measured by CCK8 after the treatment of erastin (7.5 *μ*M) or RSL-3 (0.125 *μ*M), docetaxel (D, 4 nM), and Ferrostatin-1 (Fer-1, 5 *μ*M) alone or their combination for 24 h. (e, f) After the administration of erastin (7.5 *μ*M), RSL-3 (0.125 *μ*M), and docetaxel (D, 4 nM) alone or their combined treatments for 24 h in DU145, PC3, and VCaP cells, the relative contents of ROS, MDA, and GSH were examined by commercial kits. Three independent experiments were performed. Data are reported as mean ± SD. ^∗^*P* < 0.05.

To further evaluate the impact of ferroptosis on the effect of docetaxel, PCa cells were exposed to different doses of its inducers, erastin, and RSL3. The results indicated that both of them significantly inhibited the growth of cells (Figures 2(a) and 2(b)). Furthermore, the combination of either erastin or RSL-3 and docetaxel led to a stronger cell growth inhibitory effect than a single drug in DU145, PC3, and VCaP cells, which could be partially reversed by Fer-1 treatment. However, similar effects were not found in LNCaP, C4-2B, and 22rv1 (Figures 2(c) and 2(d)). Additionally, the combined treatment resulted in an obvious increase in ROS and MDA and a higher decrease in GSH in PC3 and VCaP (Figures 2(e) and 2(f)). Together, these results indicate that ferroptosis induction improves sensitivity to docetaxel in PCa cells.

### 3.3. Ferroptosis Resistance Is Associated with Docetaxel Insensitivity in Resistant PCa Cells

**Figure 3:**
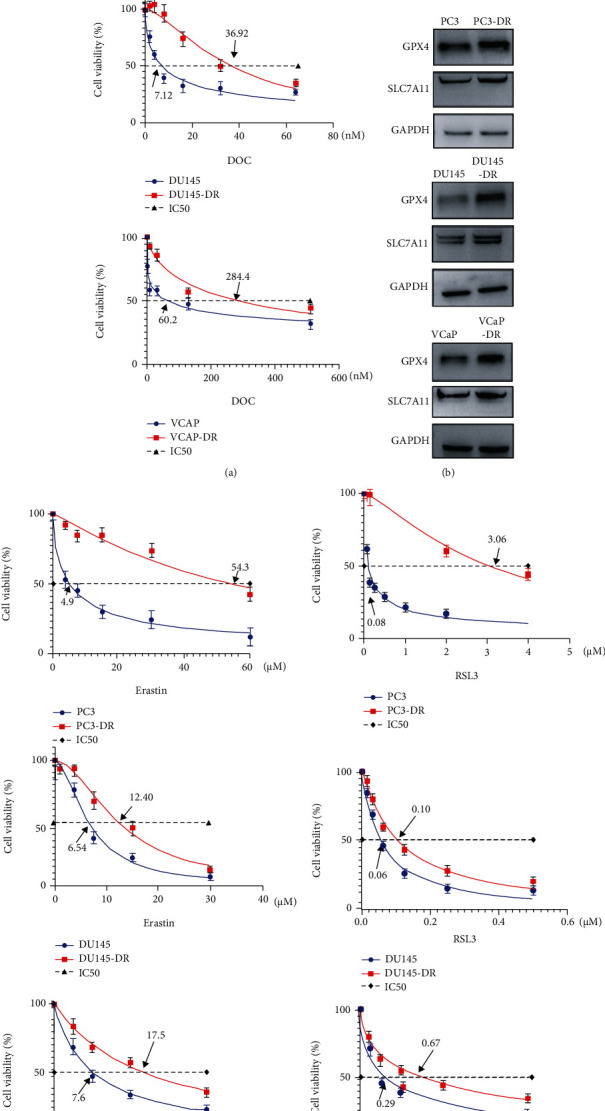
Ferroptosis resistance is associated with docetaxel insensitivity in resistant PCa cells. (a, c, d) PC3, DU145, and VCaP and their resistant cells (PC3-DR, DU145-DR, and VCaP-DR) were treated with the indicated concentrations of docetaxel, erastin, and RSL-3, respectively, for 72 h. Cell viabilities were analyzed by CCK8 assay. Data of three independent experiments were shown as mean ± SD. The dose-effect curves and IC50 values are shown. (b) The protein level of GPX4 and SLC7A11 was determined by western blotting in PC3, DU145, and VCaP and their resistant cells. Experiments were performed in triplicate; representative images are shown.

Three docetaxel resistant PCa cell lines (PC3-DR, DU145-DR, and VCaP-DR) were established in our previous study, and their resistance was also confirmed in this study based on the IC50 (half maximal inhibitory concentration) (Figure 3(a)). First, our results revealed that a marked increase in GPX4 and SLC7A11 protein levels in resistant cells was observed as compared with parental cells (Figure 3(b)). Next, we treated resistant and parental cells with different doses of erastin and RSL3. Consistent with the tolerance toward docetaxel, PC3-DR, DU145-DR, and VCaP-DR showed more resistance to erastin with the IC50 value of 54.3 *μ*M, 12.4 *μ*M, and 17.5 *μ*M, while PC3, DU145, and VCaP were more sensitive to erastin with a lower IC50 value of 4.9 *μ*M, 6.5 *μ*M, and 7.6 *μ*M (Figure 3(c)). Additionally, the IC50 of RSL3 obviously differed between PC3-DR, DU145-DR, VCaP-DR, and their parental lines (3.06 *μ*M vs. 0.08 *μ*M, 0.01 *μ*M vs. 0.06 *μ*M, and 0.67 *μ*M vs. 0.29 *μ*M), respectively (Figure 3(d)). These data indicate that docetaxel resistance is accompanied by ferroptosis tolerance in PCa.

### 3.4. Erastin and RSL3 Promote the Sensitivity of Docetaxel in Resistant Prostate Cells

**Figure 4:**
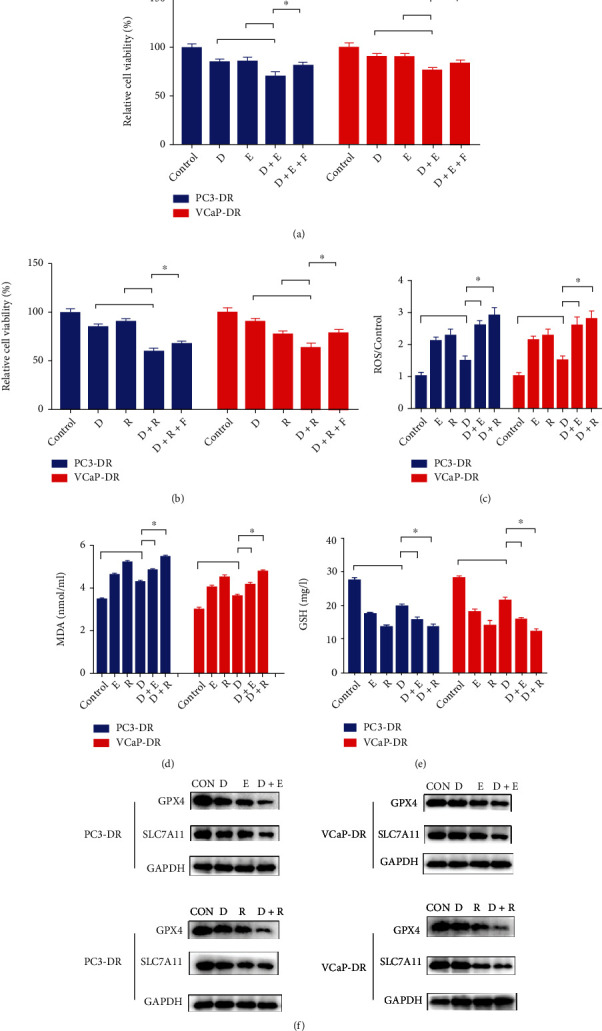
Erastin and RSL3 promote the sensitivity of docetaxel in resistant prostate cells cells. (a) The resistant cells (PC3-DR and VCaP-DR) were treated with erastin (E, 15 *μ*M) and docetaxel (D, 16 nM) alone or in combination for 48 h. (b) PC3-DR and VCaP-DR cells were treated with RSL3 (R, 0.5 *μ*M) and docetaxel (D, 16 nM) alone or in combination for 48 h. CCK8 was used to assess cell viability (a, b). (c–e) The relative contents of ROS, MDA, and GSH were detected by commercial kits, respectively. (f) PC3-DR and VCaP cells were exposed to erastin (E,15 *μ*M) or RSL3 (R,0.5 *μ*M) alone or in combination with 8 nM docetaxel for 24 h; the protein level of GPX4 and SLC7A11 was analyzed by western blotting. The data were reported as mean ± SD (*n* = 3), and representative images are shown. ^∗^*P* < 0.05.

We further examined the sufficiency of erastin and RSL3 in combination with docetaxel in PC3-DR and VCaP-DR cells. The results indicated that the combination of erastin and docetaxel dramatically decreased cell proliferation when compared with either agent alone, which could be reversed by Fer-1 (Figure 4(a)). A similar trend was observed after the combined treatment of RSL3 and docetaxel (Figure 4(b)). Coadministration of either erastin or RSL3 and docetaxel led to a significant increase in ROS and MDA content with a decrease in GSH level when compared with a single drug (Figures 4(c)–4(e)). Additionally, the cotreatment group showed more decrease in GPX4 and SLC7A11 protein in PC3-DR and VCaP-DR cells as compared to the docetaxel group (Figure 4(f)). These results demonstrate that erastin and RSL-3 can restore the sensitivity of docetaxel in resistant PCa cells.

### 3.5. Combination of RSL3 with Docetaxel Impedes Tumor Cell Growth In Vivo

**Figure 5:**
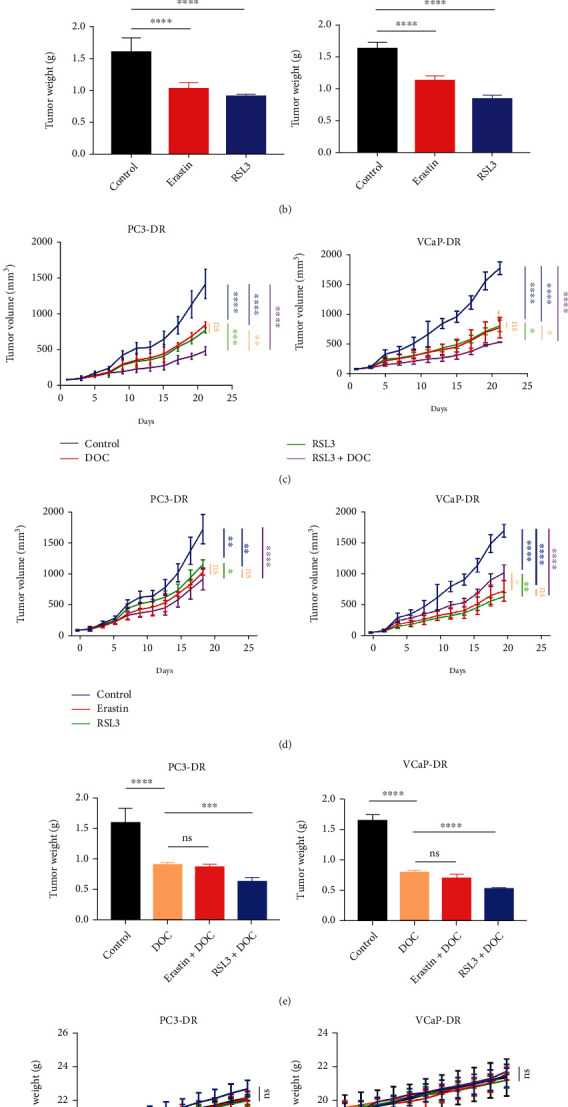
Combination of RSL3 with docetaxel impedes tumor cell growth in vivo. (a, b) Mice were transplanted with the PC3-DR and VCaP-DR cells (1 × 10^6^). Tumor-bearing mice were randomized into vehicle, erastin (E, 20 mg/kg, i.p., daily), and RSL3 (R, 100 mg/kg, i.p., biweekly). Tumor growth curve of mice for 21-day treatment of tumor (a). Tumor weights (g) were measured after tissue resection at the experimental endpoint (b). (c–e) The transplanted xenogeneic models were randomized into vehicle, docetaxel (10 mg/kg, i.p., biweekly), erastin (20 mg/kg, i.p., daily) + docetaxel (10 mg/kg, i.p., biweekly), and RSL3 (100 mg/kg, i.p., biweekly) + docetaxel (10 mg/kg, i.p., biweekly); tumor volume (c, d) and weight (e) were measured. (f) Animal weights were measured every 3 days over the treatment course and plotted. ^∗^*P* < 0.05, ^∗∗^*P* < 0.01, ^∗∗∗^*P* < 0.001.

To test the therapeutic efficacy of FINs in vivo, we established subcutaneous xenograft tumor models of PC3-DR and VCaP-DR. Administration of both erastin and RSL3 resulted in a marked suppression in tumor size when compared with the control group (Figure 5(a)). Consistently, treatment with them significantly reduced tumor weight at the endpoint (Figure 5(b)). Furthermore, the combination of RSL-3 and docetaxel decreased tumor size and weight in tumor models of PC3-DR and VCaP-DR dramatically, compared with either drug alone. However, a similar effect was not found in the combined treatment with erastin and docetaxel in the PC3-DR and VCaP-DR xenograft model (Figures 5(c)–5(e)). Importantly, we did not find any measurable side effects as assessed by animal body weight (Figure 5(f)) and signs of distress in any of the treatments when compared with vehicle control. These in vivo results further partially support the in vitro evidence that FINs could effectively enhance the anticancer activity of docetaxel by induction of ferroptosis.

## 4. Discussion

Although docetaxel is a first-line chemotherapy agent for advanced PCa, its drug resistance seriously affects its clinical efficacy. In this study, we found that ferroptosis was involved in the cytotoxic effects of docetaxel, and tolerance to ferroptosis promoted docetaxel resistance in PCa. We also provided the evidence that two frequently used FINs, erastin and RSL3, improved the efficacy of docetaxel in several PCa cells and reversed docetaxel resistance in resistant PCa cells. In vivo studies further confirmed that administration of RSL3 significantly increased the inhibitory effect of docetaxel on resistant tumors. Taken together, our study suggested the role of ferroptosis in docetaxel chemotherapy and the potential of its inducers, erastin, and RSL3, to reverse drug resistance in PCa.

Ferroptosis, a newly recognized cell death mode, is biochemically and genetically distinct from other forms of cell death and has emerged to be closely associated with cancer biology [17]. Although some studies have indicated that ferroptosis is related to the therapeutic effect of advanced PCa [14, 15, 18, 19], the influence on the chemotherapeutic effect of docetaxel is still elusive. In this present study, we found the ferroptosis inhibitor, Fer-1, could obviously attenuate the cytotoxic effect of docetaxel in several PCa cells, indicating its involvement in the process of chemotherapy. GPX4 and SLC7A11 are considered as key factors that prevent the occurrence of ferroptosis. After the treatment of docetaxel, their protein expression was found to be inhibited in a time- and concentration-dependent manner. Abnormal ROS generation and loss of GSH can trigger cell dysfunction and death, which are considered as key ferroptosis processes [20, 21]. In this study, the increased concentrations of ROS and MDA and reduced levels of GSH were observed after exposure to docetaxel, which was significantly reversed by Fer-1. Additionally, the above effects of docetaxel could be further enhanced by erastin and RSL-3. All these results suggest that ROS-mediated ferroptosis is involved in the cytotoxicity effect of docetaxel in PCa.

Currently, there are no effective treatments to reverse the chemoresistance of docetaxel in PCa, which seriously affects its clinical application effect, and thus, exploring novel potential mechanisms and therapeutic approaches for advanced PCa is very essential. A growing number of studies confirmed that ferroptosis insensitivity contributed to chemotherapy resistance in some cancers [10–13, 22]. Inducing ferroptosis has also been demonstrated to reverse the resistance of temozolomide in glioblastoma [23], cisplatin in lung cancer [24], 5-fluorouracil in colorectal cancer [25], and gefitinib resistance in head and neck cancer [26]. Consistent with previous studies, our results indicated that GPX4 and SLC7A11 protein levels were obviously elevated in docetaxel-resistant cells compared to their parental cells. In addition, resistant cells with higher IC50 to erastin and RSL-3 showed stronger tolerance to ferroptosis. Moreover, two classical ferroptosis inducers, erastin and RSL-3, markedly inhibited the growth of resistant tumor cells in vitro and in vivo. Therefore, our data indicate that ferroptosis may participate in docetaxel resistance of PCa.

Finding suitable combination therapy is an effective way to reduce docetaxel resistance and side effects. Recent studies have confirmed that a variety of FDA-approved drugs, such as flubendazole [20], brequinar [6], and sulfasalazine [27], can play an antitumor role by inducing ferroptosis, indicating the feasibility of such drugs in combination with chemotherapy. Previous studies have shown that FINs can improve the sensitivity of a variety of chemotherapeutic drugs including docetaxel [28], cisplatin [29], and oxaliplatin [30]. A recent study has confirmed that FINs were found to significantly improve the efficacy of antiandrogenic drugs for PCa without increasing side effects [15]. Another study also reported that RSL3 enhances the antitumor effect of cisplatin on PCa cells [31]. In our study, in vitro experiments showed either erastin or RSL3 in combination with docetaxel led to a stronger ferroptosis effect and improved the sensitivity to docetaxel in resistant cells. Consistently, in vivo studies further confirmed the combined inhibitory effect of RSL3 and docetaxel on resistant tumors. However, a similar effect was not observed in the combination of erastin and docetaxel, suggesting the potential role of erastin in docetaxel resistance needs deeper investigation in PCa. Thus, the above findings preliminarily demonstrated that the induction of ferroptosis can partially reverse docetaxel resistance in PCa. However, further in-depth studies are necessary to verify their effect in clinical samples.

Several recent studies have examined the molecular mechanisms that are involved in docetaxel-induced ferroptosis. In ovarian cancer, ferroptosis inducer erastin was proved to dominantly elevate the intracellular ABCB1 levels by restricting its drug-efflux activity, thus reversing ABCB1-mediated docetaxel resistance [28]. Recent research indicated that erastin could suppress the transcriptional activities of androgen receptors to promote the growth inhibitory efficacy of docetaxel on PCa cells [32]. Another recent study demonstrated that reducing iron accumulation conferred to ferroptosis inhibition and subsequently decreased docetaxel sensitivity in PCa cells [33]. In the present study, we found docetaxel-induced ferroptosis mainly by inhibiting the levels of GPX4 and SLC7A11 and promoting the accumulation of ROS. However, the exact mechanism needs to be further implemented in future research.

## 5. Conclusions

In summary, our in vitro experiments demonstrate that docetaxel induces ferroptotic cell death, and ferroptosis resistance contributes to docetaxel insensitivity in PCa cells. Erastin or RSL3 significantly improves the cytotoxic effect of docetaxel in resistant cells and nonresistant cells. Furthermore, erastin or RSL3 alone significantly delays tumor growth, and RSL3 improved the inhibitory effect of docetaxel on tumor growth of PC3-DR and VCaP-DR xenografts. Overall, although more in-depth research needs to be further carried out, preliminary experiments suggest that ferroptosis induction may represent a promising therapeutic strategy to overcome docetaxel resistance in PCa.

## Data Availability

All raw data supporting the conclusions of this study will be available from the authors upon reasonable request.
